# Supplementary figures and images for: Identification of 3-Aryl-1-benzotriazole-1-yl-acrylonitrile as a Microtubule-Targeting Agent (MTA) in Solid Tumors
Source: Int J Mol Sci. 2024 May 24;25(11):5704. doi: 10.3390/ijms25115704 (PMC11172098; doi:10.3390/ijms25115704)

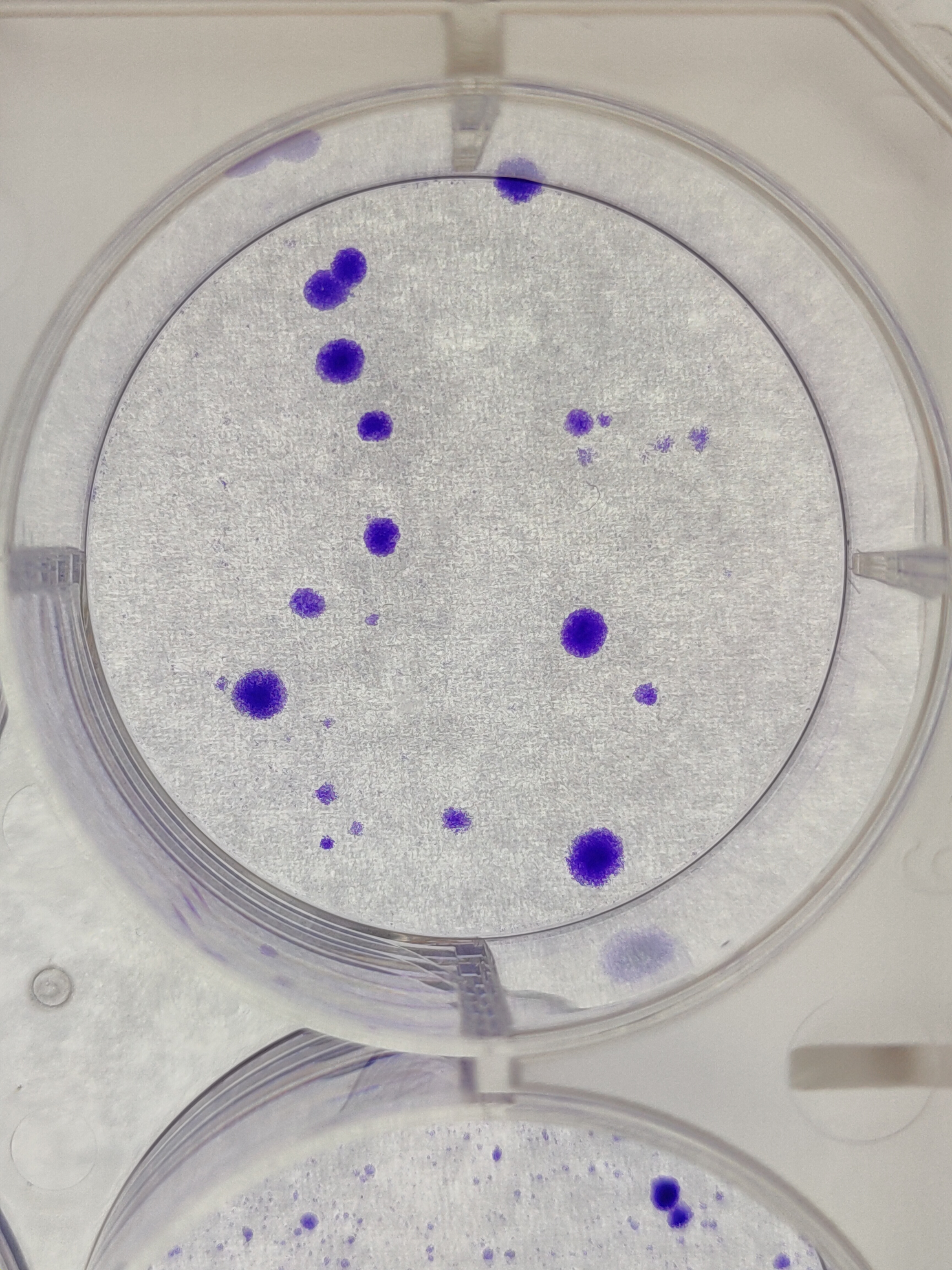

Supplement: Supplementary file 1 [file ijms-25-05704-s001.zip › 20nM.jpg]

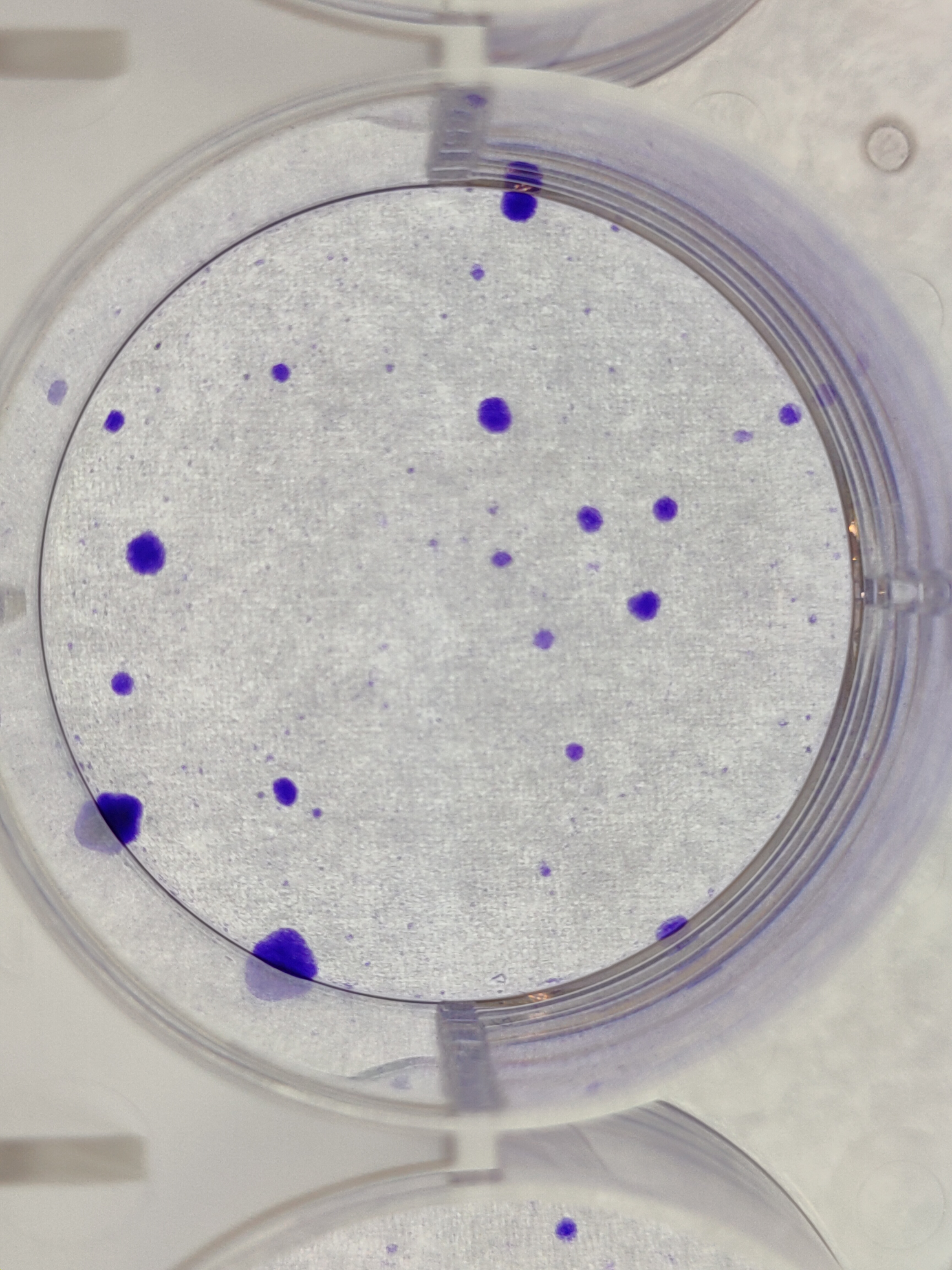

Supplement: Supplementary file 1 [file ijms-25-05704-s001.zip › 40nM.jpg]

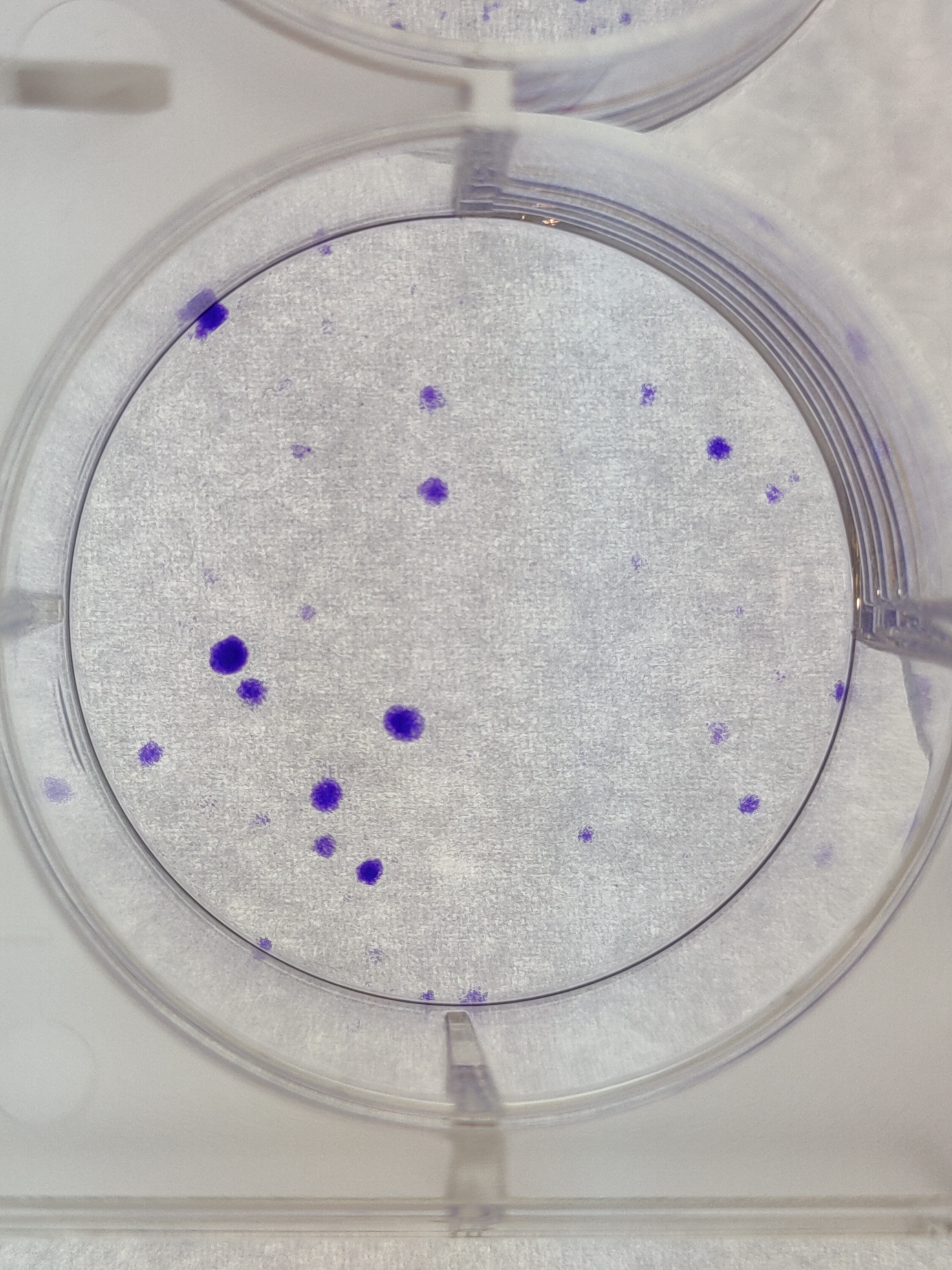

Supplement: Supplementary file 1 [file ijms-25-05704-s001.zip › 80nM.jpg]

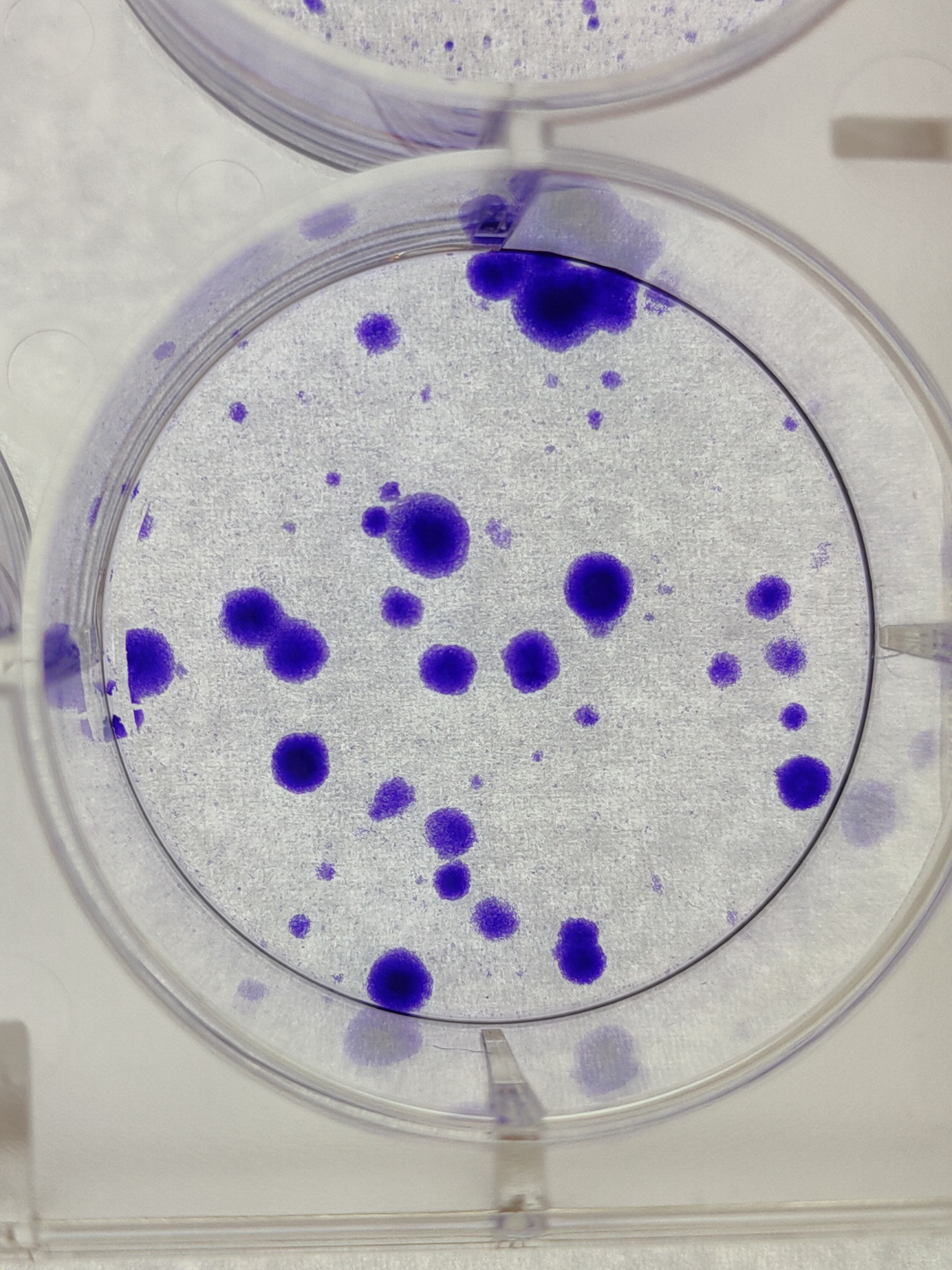

Supplement: Supplementary file 1 [file ijms-25-05704-s001.zip › CTR-.jpg]

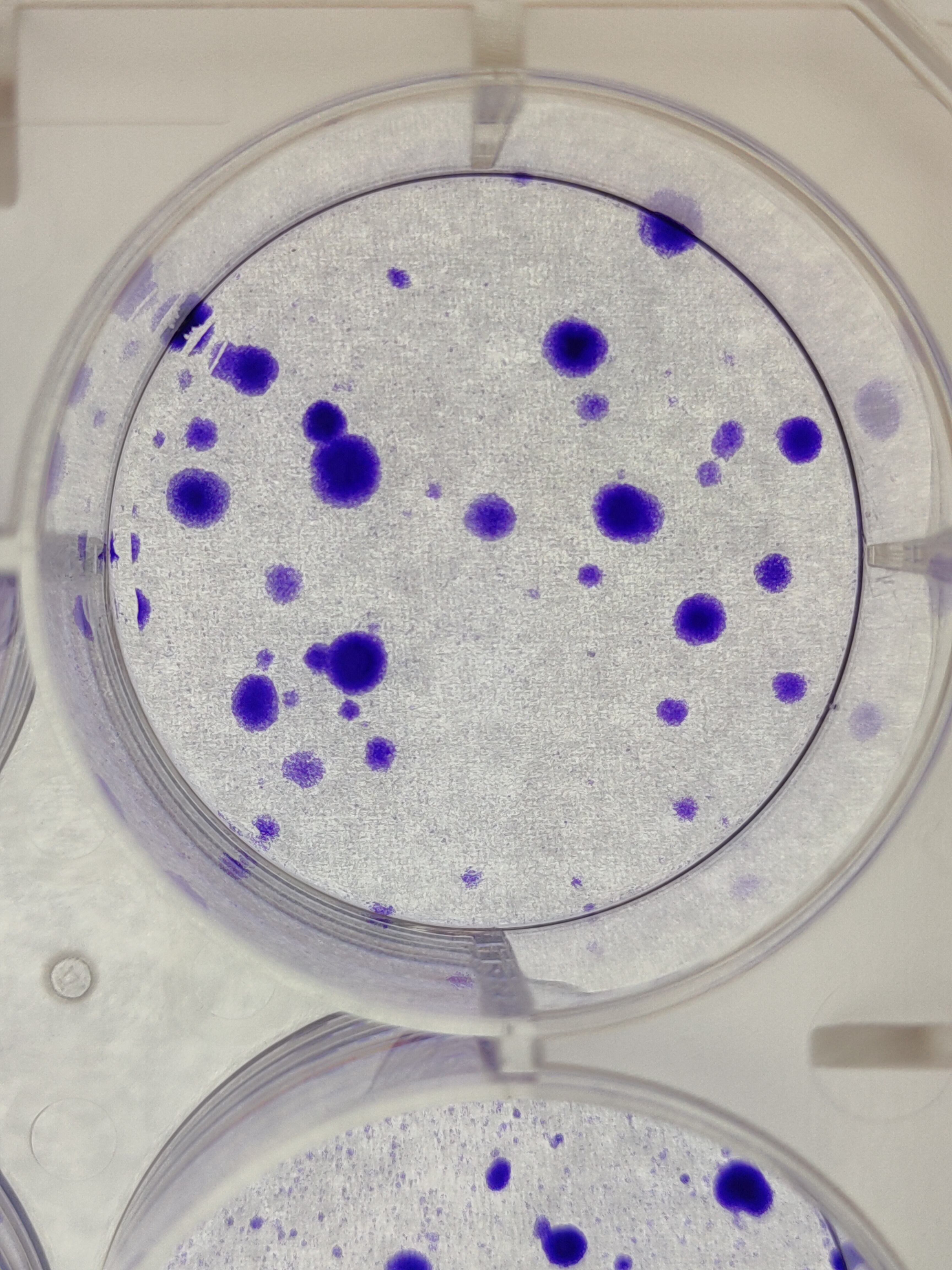

Supplement: Supplementary file 1 [file ijms-25-05704-s001.zip › DMSO.jpg]
